# Supplementary material for: The influence mechanism of work–family conflict on employees’ proactive behavior: A moderated mediator model
Source: PLoS One. 2025 Apr 30;20(4):e0321196. doi: 10.1371/journal.pone.0321196 (PMC12043168; doi:10.1371/journal.pone.0321196)
Supplement: Attachment — Submitted filename: Response to Reviewers 1230.docx [file pone.0321196.s003.docx]

Please review your reference list to ensure that it is complete and correct. If you have cited papers that have been retracted, please include the rationale for doing so in the manuscript text, or remove these references and replace them with relevant current references. Any changes to the reference list should be mentioned in the rebuttal letter that accompanies your revised manuscript. If you need to cite a retracted article, indicate the article’s retracted status in the References list and also include a citation and full reference for the retraction notice.

We would like to thank the experts for reviewing the article in their busy schedules. The research team has carefully checked the citations of the article and the literature cited in the article is complete with no retracted articles cited.

Reviewer #2:

1. Typing and language errors persist. Please go through the whole document carefully. Such as, after reference [19], word " eployees" is there.

The language throughout the text was carefully revised and checked to ensure that there were no grammatical or spelling errors.

2."Some of the items were inconvenient to be expressed in one item Control variables" is very ambigous. What does that mean?

This was a linguistic error, and the relevant content has been rephrased, as detailed in section 3.3 of the article.

3.G.power and sample validation is still missing.

Thank you very much to the experts for taking time out of their busy schedules to view my paper and now some explanations on the relevant content. The research in this paper uses SPSS and MPLUS software to analyze the data, plus software has no special requirements for sample data, and the article uses structural equation modeling to analyze the data, there is no need for sample size estimation. In addition, the sampling of data for this questionnaire survey meets the needs of the research, and the relevant information has been explained in the article. After intense discussion with the research team, there is no need to use G Power analysis or sample validation.

4. Standard error is not required for normality.

The reference to standard errors was deleted.

5.Provide IRB statement and letter of consent

Submitted IRB statement and letter of consent in the submission system, in the supporting document, titled: plos one ethics approval. and rephrased the relevant content in 3.1 Research Methodology of the article.

Reviewer #3: Thank you for addressing most of my comments. I hope the revisions improve your manuscript. Below are some additional suggestions that may further enhance your work:

1. It is recommended to refer to studies as citations in "5.1 Main Conclusions and Contributions." For instance, in the statement, “Different from the previous studies on employee proactive behavior from the perspective of social…”, and in “Third, in the existing studies on the high-commitment work system…”, you should cite the relevant research to substantiate your arguments.

Relevant references have been added to support these two references, see references 60 and 61 for details.

2. Please resolve instances of "Error! Reference source not found." in the manuscript to ensure all citations are correctly formatted.

Changes were made to the citation formatting throughout the text.

3. Certain sentences require grammatical corrections for improved readability. For example:

- “In order to test hypothesis 3 and 4. In this study, Mplus software was used to analyze the data.”

- “Emotional exhaustion (EE) is the intermediary variable at the individual level, and employee active behavior (PB) is the dependent variable at the individual level for SEM path analysis, as shown in the following table ”

- “The reliability analysis results are shown in Table 1.” it should be Table 3.

We thank the experts for carefully reading and reviewing the article in their busy schedules, and the research team members have confidently read and revised the presentation of the whole article to ensure the accuracy of the expression.

4. You mentioned: (“Control variables according to the demographic characteristics variables proposed by previous studies that may be related to the subject of this study to some extent, this paper mainly selects employee Gender, Age, Education, and LNTenure as the control variables.”)In this case, these variables should be analyzed as control variables in a manner consistent with other variables in your analysis.

Relevant analyses of control variables have been added, as detailed in section 4.5 of the text.

5. you stated that :"this paper systematically reviews the literature,".. If your paper includes a systematic literature review, consider illustrating the review process using the PRISMA criteria for clarity and methodological rigor.

The lack of a systematic literature review in this paper was an expression error, and the relevant formulation has been revised, as detailed in the first sentence of 3.3.

6. Throughout the manuscript, there are grammar, punctuation, and wording errors. It is highly recommended to have the manuscript reviewed and proofread by a native English language expert.

The full text has been revised to ensure that the manuscript matches the English context and expression.
