# Supplementary material for: The influence mechanism of work–family conflict on employees’ proactive behavior: A moderated mediator model
Source: PLoS One. 2025 Apr 30;20(4):e0321196. doi: 10.1371/journal.pone.0321196 (PMC12043168; doi:10.1371/journal.pone.0321196)
Supplement: Attachment — Submitted filename: Response to Reviewers.docx [file pone.0321196.s004.docx]

**A rebuttal letter of “The Influence Mechanism of Work-Family Conflict on Employees' Proactive Behaviors-Moderated Mediator Model”**

Dear PLoS ONE Publisher, Editor, and Reviewers：

I would like to express my sincere gratitude to the reviewers and experts for their valuable comments on the article "The Influence Mechanism of Work-Family Conflict on Employees' Proactive Behaviors-Moderated Mediator Model". Based on the comments and suggestions of the reviewers, the author has made revisions to the article, and I will explain each revision in turn below:

Reviewer #2: Dear author,
Please add rationale for not mentioning the minimum sample size for structual equtation modelling with citation.Kindly, draw on more detail for sampling. The inclusion criteria needs to be added for companies/enterprises and teams.Please eradicate any casual pharases like " so on"

Thanks for the suggestions of the reviewers. After discussion by the research team, the sample size calculation and effect size analysis of the data have been carried out based on the opinions of the experts. See 4.1 in the article for details. The sample size is sufficient. Firstly, theoretically, there is a close logical connection between the independent variables (work-family conflict, emotional exhaustion and high-commitment work system) and the dependent variable (proactive behavior of employees). According to previous studies, the three independent variables may have a substantial impact on the dependent variable. Secondly, from the perspective of the measurement quality of each variable, the study uses multiple maturity scales, and the α coefficients of the four variables of work initiative behavior, work-family conflict, emotional exhaustion and high-commitment work system are 0.932, 0.912, 0.916 and 0.908 respectively, all higher than the excellent level of 0.90. This shows that the internal consistency of the four scales is very high, and the measurement results are highly reliable. Each item has a strong correlation with the dimension, which can stably and reliably reflect the individual differences of the corresponding constructs. Good measurement quality provides an important guarantee for further investigation of the substantive relationship between variables. Based on the accumulation of theoretical and empirical studies, it is reasonable to select a large effect size f²=0.35, which not only conforms to theoretical expectations, but also has a good measurement basis. Below is the output graph of G power.

Reviewer #3: I strongly advise the authors to check the manuscript with a native proofreader as it has a lot mistakes. These are some:
Title: Consider using a colon (:) instead of a dash (–) before "A Moderated Mediator Model" to align with proper title formatting.
Keywords: Ensure consistency in capitalization.
Introduction: Rewrite the phrase "businesses-focused HRM tactics" to include the full term before introducing the abbreviation for HRM.
Literature Review: Include the year of publication in parentheses after mentioning the authors. For example:
• "According to empirical research, Xu Yan (XXXX) discovered that work-family conflict has a significant negative impact on employees' work performance."
• "Chinese scholar Li Yifei et al. (XXXX) discovered through empirical analysis that work-family conflict negatively affects job satisfaction."
• "Zhang Junwei et al. (XXXX), using the resource conservation theory, found that work-family conflict significantly affects performance."
• Similarly, for Min's research, add: "Min (XXXX) stated that a high-commitment work system encourages initiative and creativity while reducing negative emotions."

2.2 The Mediating Effect of Emotional Exhaustion on Employees' Proactive Behavior: Ensure the indentation format of this section is consistent with other subheadings in the manuscript.
Clarifications and Edits:
• Proofread several sentences such as: " A person who experiences negative emotional reactions will be less enthusiastic and have a worse attitude at work, which will lead to burnout and a loss of commitment to the company. could possibly trigger the trend toward turnover [34].
• Replace "data" with appropriate word in: "In light of this data, we put forth the following hypothesis."
• Improve punctuation and flow, for example in: " putting in place employee involvement plans and flexible work designs to encourage diverse communication and hearing what staff members have to say [40] Employees are viewed as partners in the high-commitment work system, and the company can truly understand their requirements and communicate the corporate goal to them through active communication and other channels. to be acknowledged by workers and, via emotional dedication, to achieve the shared objectives and values of the company and workers."
• Replace "theories" with "hypotheses" in: "In light of this, the following hypotheses are put forth."
• Rephrase the paragraphs to place the hypothesis statement at the end. For example, in the sentence: " Hypothesis 3: The impact of emotional weariness on proactive conduct by employees is negatively moderated by a high-commitment work structure. This means that companies that have adopted a high-commitment work system have less of an effect on employees' proactive behavior than companies that have not adopted one or that have low levels of commitment in their HRM practices."
Clarifications on Terminology:
Clarify whether you are referring to "theory" or "hypothesis" in all applicable sentences, for example: " In addition to the aforementioned theory, we propose that the degree of high-commitment work systems that firms have in place may also have an impact on how work-family conflict affects employees' active behavior through emotional weariness."
And proofread and rephrase this sentence for clarity and accuracy: "Establishing a high-commitment work system can provide employees with human capital (skills and knowledge needed for tasks), emotional resources (positive emotional states), and social resources (a sense of security, accomplishment, and self-realization), encouraging productive behaviors that benefit the organization."
And Clarify the term "LNTenure."
And The sign "—" (dash) appears frequently in the manuscript. Proofread and replace it with a consistent and appropriate symbol.
4.5 Hypothesis Testing: Use plural form (e.g., "Hypotheses Testing") for this section title.
Tables: Ensure all tables are properly aligned and formatted to fit within the page, such as Table 12 and Table 13.

Citations: When mentioning "studies" or "research," always include proper citations to support the statements.
Variables items: Please list all items or state sample for each variable for items that you used in your adapted/adopted items, for clarity and credibility

Thank you very much for taking time out of your busy schedule to read and review this article. The problems you mentioned have been revised one by one and marked in the revised draft.
